# Temperature and precipitation in the US states: long memory, persistence, and time trend

**DOI:** 10.1007/s00704-022-04232-z

**Published:** 2022-10-29

**Authors:** Luis A. Gil-Alana, Rangan Gupta, Laura Sauci, Nieves Carmona-González

**Affiliations:** 1 University of Navarra (Faculty of Economics, ICS and DATAI), 31080 Pamplona, Spain; 2 Universidad Francisco de Vitoria, Madrid, Spain; 3 University of Pretoria, Pretoria, South Africa; 4 International University of La Rioja, Logroño, La Rioja Spain

## Abstract

This paper investigates the time series properties of the temperature and precipitation anomalies in the contiguous USA by using fractional differentiation. This methodology allows to capture time trend components along with properties such as long-range dependence and the degree of persistence. For aggregated data, we find out that long memory is present in both precipitation and temperature since the integration order is significantly positive in the two cases. The time trend is also positive, being higher for the temperature. In addition, observing disaggregated data by states, for the temperature, there are only seven states where the time trend is not significant, with most of them located in Southeast areas, while for the rest of cases, the time trend is significantly positive. All cases exhibit long-range dependence, though the differencing parameter substantially changes from one state to another, ranging from 0.09 in Nebraska and Kansas to 0.18 in Florida and Michigan. For precipitation, the time trend is insignificant in a large number of cases, and the integration order is smaller than for the temperature. In fact, short memory cannot be rejected in fourteen states, and the highest orders of differencing are obtained in Arizona (*d* = 0.11) and Texas (0.12). In general, we highlight that one cannot draw conclusions about persistence and trends in these two climate-related variables based on aggregate information of the overall USA, given widespread heterogeneity across the states. Tentatively, the degree of dependence across the states seems to be negatively correlated with their level of climate-related risks and the associated preparedness in terms of handling climate change, but this conclusion requires more elaborate research in the future.

## Introduction

The analysis of climate variability is essential to make reliable long-term predictions. According to NOAA National Centers for Environmental Information, 2021 was ‘the fourth-warmest year in the 127-year record’ for the contiguous US, estimating a warming trend of + 1.60ºF/100 years and a precipitation trend of + 1.88in/100 yr (NOAA National Centers for Environmental information, 2022). In addition, it was detected extreme atmospheric events -wet and dry- in that region for the same year. In this context, the Palmer Drought Severity Index (PDSI) showed a trend of + 0.34/century, finding an increase in the drought risk in several areas such as in the Southwest and Southeast (Ge et al. 2016; Apurv and Cai, 2021), the Northwest and the Northern Great Plains (Ge et al. 2016).

Given that the climatological time series carry implicit long memory properties, for addressing an adequate study of climate trends, we will consider the long-range dependence of observations—or long memory processes—which implies ‘that even the most distant past still influences the current and future climate’ (Franzke et al. 2020). In this sense, the existence of a warming trend in average temperature is consistent with previous studies based on the fractional integration approach which also find significant positive trends in the Northern Hemisphere for that time scale (Gil-Alana, 2003, 2005, 2012, 2018; Gil-Alana and Sauci, 2019). Nevertheless, there is no wide consensus about whether there is persistence in the precipitation process (Yang and Fu, 2019), which seems to be dependent on the latitude, the climatical characteristics of each station, and the degree of homogeneity of the series (Potter, 1979; Tyralis et al. 2018). All this without forgetting that the long memory or long-range dependence properties may be affected by cross-sectional aggregation (Vera-Valdés, 2021), or by scales (Graves et al. 2017; Franzke et al. 2020).

The purpose of the paper is to look at the temperature and precipitation anomalies in the US, both aggregated and disaggregated by states in order to determine if there are significant time trends in the data, over the monthly period of 1895:01 to 2021:10, using a model of form as in the following equation:

1 $$\begin{array}{ccc}y_t=a+\beta t+x_t,&\left(1-B\right)^d\;x_t=u_t,&u_t=\rho\;u_{t-12}+\varepsilon_t.\end{array}$$

where y_t_ refers to the observed data; α and β are unknown parameters, namely the constant (intercept) and the linear time trend coefficient; t is a time trend; B indicates the backshift operator; d is a real value that indicates the number of differences to be adopted in x_t_ to achieve I(0) stationarity; x_t_ shows the regression errors, assumed to be thus integrated of order d or I(d), which implies that u_t_ is short memory or I(0); in addition, given the possible seasonality of the monthly series analyzed, a seasonal AR(1) process is assumed for the I(0) disturbances u_t_, where ρ is the (monthly) seasonality indicator, and ε_t_ is a white noise process.

Note that the estimation of β is crucial and it is clearly determined by the type of assumptions made of x_t_. Most articles impose *d* = 0 or alternatively *d* = 1. However, our results show that d is between 0 and 1. With our paper being the first of its kind for the aggregate US and its states over the longest possible sample period, which helps us avoid sample selection bias, our results also have novel economic implications. In fact, the novelty of this paper is more its application, and we do not aim to provide any theoretical econometric model innovation. In the paper we want to estimate time trends but simultaneously allowing for the possibility of strong dependence or long memory, noting that not taking this into account may produce inconsistent estimates of the deterministic terms.

In this regard, we use the well-established Autoregressive Fractionally Integrated Moving Average (ARFIMA) model to study long-memory persistence, and trends of long-spans of aggregate and state-level data of the US on temperature and precipitation anomaly. The objective is to highlight the heterogeneity in the underlying long-memory, persistence, and trend estimates of the aggregate and regional data to emphasize the fact that when analyzing these properties of climate-related variables of the US, we cannot generalize the findings obtained for the overall US to the regions, i.e., states. This is important since this has important implications for policymaking in terms of heterogeneous degree of responses at the state level to the issue of climate change as measured by these two variables under investigation.

The structure of this paper is as follows. Section 2 presents a brief summary of the literature. Section 3 indicates the methodology applied while Section 4 describes the dataset used, and presents the results. Finally, Section 5 discusses and concludes the paper.

## A review of the literature

According to the World Meteorological Organization (WMO 2020), there is a 20% chance that by 2024 we will exceed 1.5 °C; therefore, if the current rate of increase in greenhouse gas concentrations is maintained, the increase in temperature by the end of this century will exceed the limit established in the Paris Agreement to limit global warming to 1.5 or 2 °C above pre-industrial levels (WMO, 2021). 

Not even the industrial and economic slowdown caused by COVID-19 slows global warming as the persistence of carbon dioxide (CO_2_) in the atmosphere is very prolonged and therefore the reduction in emissions in 2021 is not likely to lead to a decrease in atmospheric concentrations of CO_2_ that drive the rise in global temperature (WMO, 2020).

The study, evaluation and trend of climate change have a great interest reflected in the numerous scientific studies (Bloomfield, 1992; Folland et al. 2018; Brunetti et al. 2001; etc.). However, there is no common criterion either on the modelling of the most appropriate climatological time series, nor on the deterministic nature of the term tendential in temperature time series.

As for modelling, one option is to consider that the time series of temperatures are stationary I(0) (Bloomfield and Nychka, 1992; Woodward and Gray, 1993) or non-stationary Models I(1) (Woodward and Gray, 1995; Stern and Kaufmann, 2000; Mann, 2004; Hamdi et al. 2018). Other studies consider wavelet analysis that allows the analysis of very large data sets being very robust against the presence of deterministic trends, in addition to allowing their detection and identification (Abry and Veitch, 1998), detrended fluctuation analysis based on a generalization of the analysis of fluctuation without trend (Kantelhardt et al. 2001) or spectral analysis where the correlations of several daily surface meteorological parameters are analyzed by partially complementary methods that are effective on different time scales (Weber and Talkner, 2001).

A common way to study the evolution of temperature is by diagnosing the nature (stochastic or deterministic) of the term trend in time series, without reaching conclusive results. While studies confirm stochastic behavior (Kallache et al. 2005; Cohn and Lins, 2005; Koutsoyiannis and Montanari, 2007; Hamed, 2008) others find a positive, deterministic, and statistically significant trend (Bloomfield and Nychka, 1992; Vogelsang and Franses, 2005; Fatichi et al. 2009).

Many studies focus on standard regressions over time trying to test whether the time trend coefficient is significantly positive and where the errors follow a short memory process or I(0).

Time series study, using power spectral density (PSD) analysis, often gives false results due to the highly non-stationary nature of rainfall signals (Matsoukas et. al. 2000; Kantelhardt et al. 2006). To avoid this, some authors have used trendless fluctuation analysis (DFA), and its multifractal generalization, the multifractal DFA (MF-DFA) (Jiang. et al. 2017; Philippopoulos et al. 2019; Kalamaras et al. 2019; Gómez-Gómez et al. 2021) and its multifractal generalization (Kantelhardt et al. 2002). Nevertheless, these techniques can lead to more variability and bias by overestimating or underestimation fractal parameters (Maraun et al. 2004; Stadnitski, 2012; Roume et al. 2019; etc.), especially in ‘short series of persistent noise’ (Delignieres et al. 2006). This could be due to the intrinsic characteristics of DFA method itself (Carpena et al. 2017) and the data transformations that need to be performed in this approach (Stadnitski, 2012).

In contrast, the main advantage offered by the autoregressive fractional integrated moving average (ARFIMA) approach (Granger and Joyeux, 1980; Hosking, 1981) is that the differentiation parameter *d* can be a real number, which allows a more accurate description of correlation not only at long-term but also at short-term (Huang et al. 2022). So, ARFIMA analysis and fractional integration in general provide efficient estimations and less variability (Roume et al. 2019; Bhardwaj et al. 2020) that could improve and complement the analysis realized by classical algorithms (Delignieres et al. 2006; Torre et al. 2007, and others).

The literature is very extensive, and the behavior of long memory in the study of temperature series should not be neglected (Lenti and Gil-Alana, 2021). In fact, long memory, and specifically fractional differentiation, has been widely used in the analysis of temperatures (Gil-Alana, 2005, 2006, 2017; Vyushin and Kushner, 2009; Zhu et al. 2010; Rea et al. 2011; Franzke, 2012; Yuan et al. 2013). Gil-Alana (2018) studied the time trend coefficients of temperatures in the US 48 states from 1895 to 2017 using techniques based on fractional integration in the untrended series. The results are more accurate trend estimates than those obtained with other methods that assume I(0) seasonality and I(1) non-seasonality.

Gil-Alana and Sauci (2019) assess the fractional persistence of average temperature and anomalies using monthly US data for the period 1895–2017. Their results show positive and significant trend coefficients for 38 out of 48 states, observing a high degree of persistence in most of the series. In particular, the states of Rhode Island, New Jersey, and North Caroline exhibit the greatest increase above 2.70 °C/100 years. The present study extends this analysis to a longer US temperature dataset and includes moreover time series of precipitation.

## Methodology

Taking into account the monthly structure of the series under examination, and in order to test both the existence of trends and the degree of dependence, we examine the model given by Eq. (1), that is, including a linear trend, an I(d) model, and a seasonal AR structure.

In this context, there are three parameters of interest, β, that indicates the increase in the value of the series per unit of time (months); *d*, referring to the degree of dependence or persistence, and showing long memory if that parameter is significantly positive; and ρ, the seasonal AR coefficient, dealing with the seasonally (monthly) structure.

Focusing on the long memory property, this is a feature of time series data that implies that observations are very dependent even if they are separated in time. Among the many models describing this type of behavior, a very common one is that based on fractional differentiation, which is described by the second equality in Eq. (1) and that satisfies this long memory property is d is positive. Being a real value, it allows us to consider different alternatives such as I(0) or short memory (if *d* = 0), stationary long memory (0 < *d* < 0.5); nonstationary though mean-reverting behavior (0.5 ≤ *d* < 1); unit roots (*d* = 1) or even explosive behaviours (*d* > 1).

The long memory feature on fractional integration can be easily seen from the Binomial expansion of (1 – B)^d^ which is:

$$\left(1-B\right)^d=\sum_{j=0}^\infty\begin{pmatrix}d\\j\end{pmatrix}\left(-1\right)^j\;B^j=1-d\;B+\frac{d\;\left(d-1\right)}2B^2-\dots$$

and thus, the higher the value of the differencing parameter *d* is, the higher the association between observations, even if they are far apart. Robinson (1978) and Granger (1980) justified the presence of long memory based on the aggregation of heterogeneous autoregressive (AR) processes, and fractional integration was first introduced in the literature by Granger and Joyeux (1980) and Hosking (1981), being widely used in the context of aggregated data since the late 90 s (Baillie, 1996; Hsueh and Pan, 1998; Gil-Alana and Robinson, 1997; Parke, 1999; etc.).

The estimation of the model is conducted by means of an approximation of the likelihood function, the Whittle function, expressed in the frequency domain, and we use a technique that is a testing method proposed in Robinson (1994) and that is very appropriate in our case, since it does not impose stationarity in the series unlike most of the classical long memory procedures.

Robinson (1994) proposes the following regression model,

2 $$\begin{array}{cc}{\mathrm y}_t=\beta^Tz_t+x_t;&t=1,\;2,\dots,\end{array}$$

where z_t_ is a (kx1) vector of exogenous regressors (or deterministic terms) and the regression errors, x_t_, are described as:

3 $$\left(1-B\right)^{d_1}\;\left(1+B\right)^{d_2}\prod\nolimits_{j=3}^m\left(1-2\;\cos\;w_r^j\;B+B^2\right)^{d_j}\;x_t=u_y,$$

where *d* is a (mx1) vector of real-value parameters, where the first component d_1_ refers to the long run or zero frequency, and the rest of the terms (*d*_j_, *j* > 1) refer to the orders of integration at non-zero frequencies. ${w}_{j}^{r}=\frac{{2\pi r}_{j}}{T}$; and ${r}_{j}=\frac{T}{{S}_{j}}.$ Thus, r_j_ refers to the frequency with a pole or singularity in the spectrum of x_t_, and s_j_ indicates the number of periods per cycle, while Robinson (1994) proposed to test the null hypothesis:

4 $${H}_{O}:d={d}_{o}$$

in the model given by (2) and (3) for any real value-vector d_o_, and he showed that the test statistics, say $\widehat{\mathrm{R}}$ has a ${\chi }_{\mathrm{m}}^{2}$-null limit distribution. In the empirical work carried out in the following section, we suppose *m* = 1, and thus, we only consider the long run or zero frequency. Thus, the limiting distribution is ${\chi }_{1}^{2}$.

## Data and empirical results

### Data

For our analyses, we use monthly data on temperature and precipitation anomalies (relative to the base period of 1901–2000) for the aggregate US and its 48 contiguous states (i.e., except for Alaska and Hawaii) over the monthly period of 1895:01 to 2021:10. The data is sourced from the National Oceanic and Atmospheric Administration (NOAA).^1^

### Aggregated data results

Table 1 presents the estimates of the integration order d in Eq. (1) for the two aggregated time series. We display the results under the three classical assumptions in the unit root literature, i.e., (i) with no deterministic components, i.e., imposing that *α* = *β* = 0 a priori in (1); (ii) including only an intercept or a constant, i.e., with *β* = 0 a priori; and (iii) including both an intercept and a (linear) time trend, i.e., with both parameters *α* and β estimated from the data. Together with the estimates of the differencing parameter d we also present in the tables the confidence intervals for the non-rejection values of *d* at the 5% level using the tests of Robinson (1994).

**Table 1:** Estimates of fractional differentiation parameter, *d* aggregated data

Series	*α* = *β* = 0 in Eq. (1)	*β* = 0 in Eq. (1)	*α* and *β* estimated from the data
Temperature anomaly	0.16 (0.13, 0.19)	0.16 (0.13, 0.19)	**0.13 (0.09, 0.16)**
Precipitation anomaly	0.13 (0.10, 0.17)	0.13 (0.10, 0.18)	**0.13 (0.09, 0.17)**

The values in boldface are the selected model. The values in parenthesis after the numbers correspond to the 95% confidence bands of non-rejections values of the integration parameter *d*

**Fig. 1:**
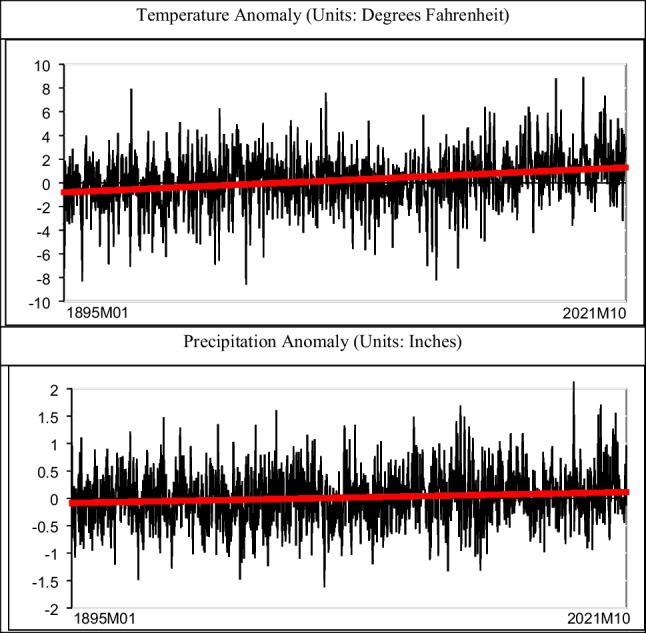
Time series plots and estimated trends

We report in boldface in Table 1 the selected model according to the best specification of the deterministic terms. This selection has been made based on the significance of the estimated coefficients in (1). Thus, if both deterministic terms, i.e., *α* and *β* are statistically significantly different from zero, we adopt that model; if β is found to be insignificant, we choose the model with only an intercept, while if both are statistically insignificant, we adopt the model with no deterministic terms. We see in the table that for the two series the time trend is required and the estimated value of *d* is 0.13 in the two series. Table 2 displays the coefficients based on the selected model. We see that the estimates of β are significantly positive in the two series, being much higher in the case of temperatures than in precipitation. The estimated time trends are graphically displayed in Fig. 1.

**Table 2:** Estimated coefficients in the regression model: aggregated data

Series	*d*	*α* (*t*-value)	*β* (*t*-value)
Temperature anomaly	0.13 (0.09, 0.16)	− 0.81125 (− 3.62)	0.00138 (5.63)
Precipitation anomaly	0.13 (0.09, 0.17)	− 0.08431 (− 1.62)	0.00013 (2.36)

A *t*-value above 1.64 in absolute value indicates support of significance of the estimated coefficient

### Disaggregated data by states

**Fig. 2:**
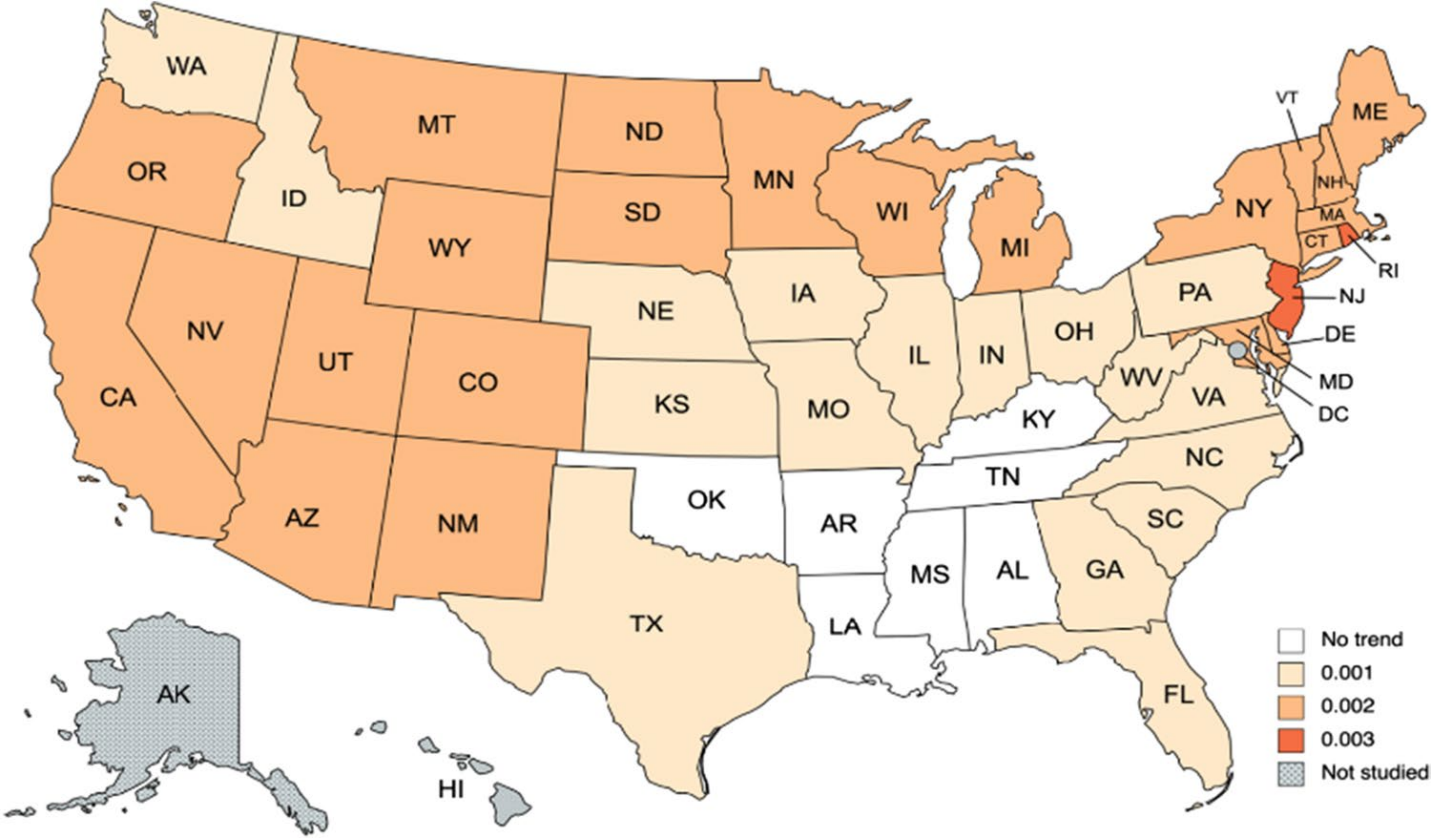
Time trend for temperature anomaly, based on results of Table 4

**Fig. 3:**
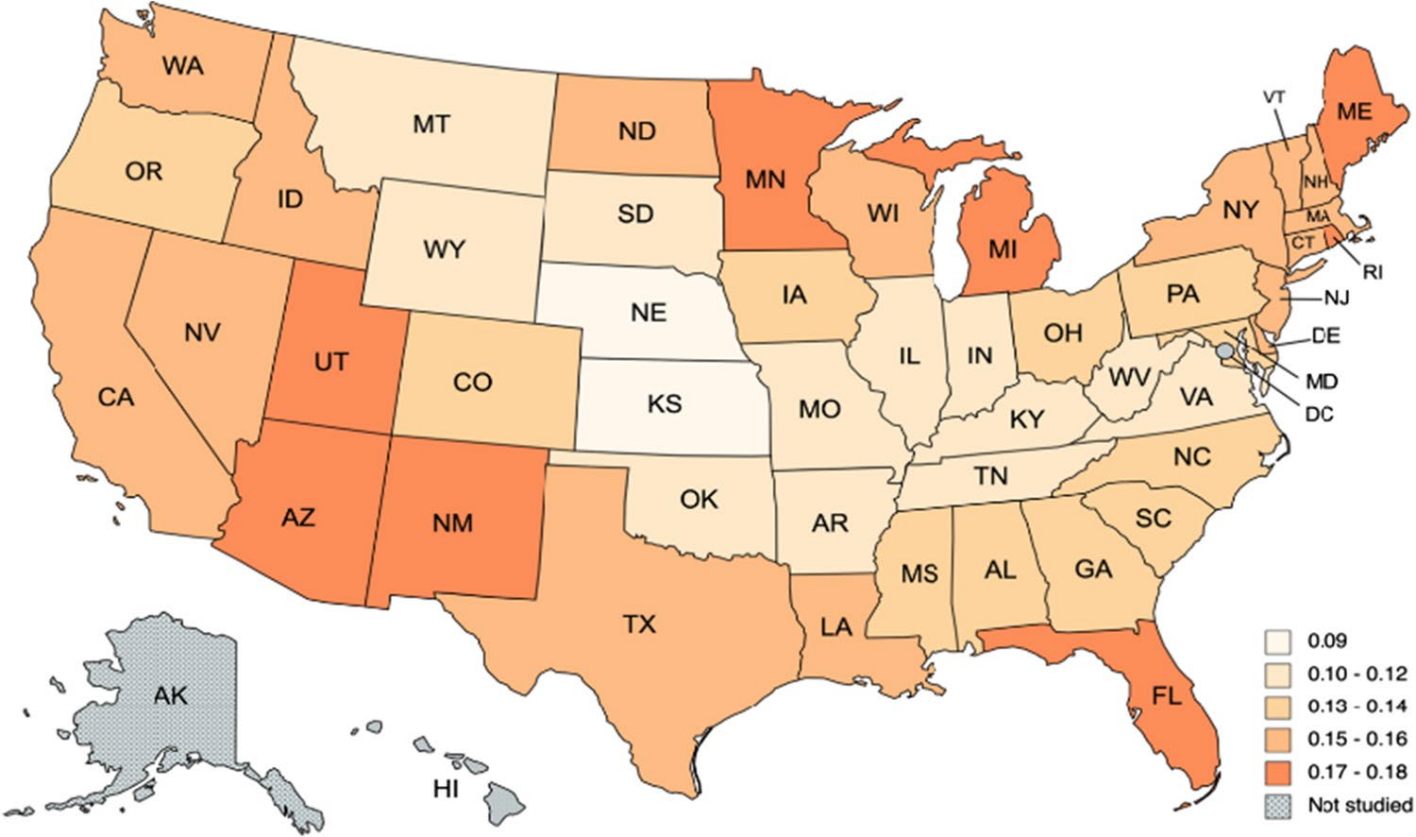
Estimate of *d* for temperature anomaly, based on results of Table 6

We start reporting the results for the temperatures (see, Tables 3 and 4). The first observation from Table 3 is that the model with a time trend is preferred in the majority of the cases. In fact, there are only seven states where the model does not require either a constant or a time trend. They are Alabama, Arkansas, Kentucky, Louisiana, Mississippi, Oklahoma, and Tennessee, which are all geographically related in the Southeastern part (see Fig. 2). Focusing on the selected models, in Table 4, we observe that the estimate of the differencing parameter is significantly positive in all cases, ranging from 0.09 (Nebraska and Kansas) and 0.10 (Missouri, Montana, Oklahoma and Wyoming) to 0.18 in Florida and Michigan. Figure 3 provides a graphical summary of the results relating the differencing parameter. Tentatively, the degree of persistence seems to be correlated in a negative manner with climate change–related risks and how prepared the states are in terms of climate change, i.e., what measures they are undertaking to slow down the process of climate change. In this regard, the reader is referred to a non-academic analysis that was conducted by a private company dealing with homeowners insurance namely Policygenius. In particular, see, https://www.policygenius.com/homeowners-insurance/best-and-worst-states-for-climate-change/. The company has developed what it calls the 2021 Policygenius Best & Worst States for Climate Change Index.^2^ To calculate this index, a ranking was provided for each of the contiguous 48 states on several climate change-related factors.^3^ Then a score out of 100 was created for each state based on these rankings. A higher score means a better outlook in a low or high-emissions future, and a lower score means a worse outlook. We observe that the lowest degrees of persistence seem to take place in the central part of the US (Nebraska and Kansas). Finally, the seasonal AR coefficient seems not to be much significant in any of the US states.

**Table 3:** Order of integration (*d*) in the temperature anomaly: results by state

Series	α = β = 0 in Eq. (1)	*β* = 0 in Eq. (1)	*α* and *β* estimated from the data
Alabama	**0.13 (0.09, 0.17)**	0.13 (0.09, 0.17)	0.13 (0.09, 0.17)
Arizona	0.20 (0.17, 0.24)	0.21 (0.17, 0.24)	**0.17 (0.14, 0.22)**
Arkansas	**0.11 (0.08, 0.15)**	0.11 (0.08, 0.15)	0.11 (0.08, 0.15)
California	0.20 (0.17, 0.24)	0.21 (0.17, 0.24)	**0.16 (0.13, 0.20)**
Colorado	0.16 (0.13, 0.20)	0.16 (0.13, 0.20)	**0.13 (0.09, 0.17)**
Connecticut	0.20 (0.16, 0.23)	0.20 (0.17, 0.23)	**0.16 (0.12, 0.20)**
Delaware	0.18 (0.15, 0.21)	0.18 (0.15, 0.21)	**0.15 (0.11, 0.19)**
Florida	0.19 (0.16, 0.24)	0.20 (0.16, 0.24)	**0.18 (0.13, 0.22)**
Georgia	0.15 (0.11, 0.19)	0.15 (0.11, 0.19)	**0.14 (0.10, 0.19)**
Idaho	0.17 (0.14, 0.21)	0.18 (0.14, 0.22)	**0.16 (0.12, 0.20)**
Illinois	0.12 (0.09, 0.15)	0.12 (0.09, 0.15)	**0.11 (0.08, 0.16)**
Indiana	0.12 (0.09, 0.17)	0.12 (0.09, 0.17)	**0.12 (0.08, 0.16)**
Iowa	0.13 (0.09, 0.18)	0.13 (0.10, 0.18)	**0.13 (0.09, 0.17)**
Kansas	0.10 (0.07, 0.14)	0.11 (0.07, 0.14)	**0.09 (0.06, 0.13)**
Kentucky	**0.12 (0.08, 0.16)**	0.12 (0.08, 0.16)	0.11 (0.08, 0.15)
Louisiana	**0.15 (0.11, 0.19)**	0.15 (0.11, 0.19)	0.15 (0.11, 0.19)
Maine	0.21 (0.18, 0.25)	0.21 (0.18, 0.25)	**0.17 (0.13, 0.22)**
Maryland	0.16 (0.13, 0.20)	0.17 (0.13, 0.20)	**0.14 (0.10, 0.18)**
Massachusetts	0.20 (0.17, 0.23)	0.20 (0.17, 0.23)	**0.16 (0.13, 0.20)**
Michigan	0.20 (0.16, 0.24)	0.20 (0.17, 0.24)	**0.18 (0.14, 0.22)**
Minnesota	0.18 (0.14, 0.22)	0.18 (0.15, 0.22)	**0.17 (0.12, 0.21)**
Mississippi	**0.13 (0.10, 0.17)**	0.13 (0.10, 0.17)	0.13 (0.10, 0.17)
Missouri	0.10 (0.07, 0.14)	0.10 (0.07, 0.14)	**0.10 (0.06, 0.14)**
Montana	0.12 (0.09, 0.16)	0.13 (0.09, 0.16)	**0.10 (0.06, 0.14)**
Nebraska	0.11 (0.07, 0.14)	0.11 (0.07, 0.14)	**0.09 (0.05, 0.13)**
Nevada	0.18 (0.14, 0.22)	0.18 (0.15, 0.22)	**0.15 (0.11, 0.20)**
New Hampshire	0.19 (0.16, 0.23)	0.19 (0.16, 0.23)	**0.16 (0.13, 0.20)**
New Jersey	0.20 (0.17, 0.23)	0.20 (0.17, 0.23)	**0.15 (0.12, 0.20)**
New Mexico	0.20 (0.16, 0.23)	0.20 (0.17, 0.23)	**0.17 (0.14, 0.21)**
New York	0.17 (0.13, 0.21)	0.17 (0.14, 0.21)	**0.15 (0.11, 0.19)**
North Caroline	0.14 (0.10, 0.17)	0.14 (0.10, 0.18)	**0.13 (0.09, 0.17)**
North Dakota	0.16 (0.13, 0.21)	0.17 (0.13, 0.21)	**0.15 (0.11, 0.19)**
Ohio	0.13 (0.10, 0.17)	0.14 (0.10, 0.18)	**0.13 (0.09, 0.17)**
Oklahoma	**0.10 (0.07, 0.14)**	0.10 (0.07, 0.14)	0.10 (0.06, 0.14)
Oregon	0.18 (0.15, 0.22)	0.18 (0.15, 0.22)	**0.14 (0.10, 0.19)**
Pennsylvania	0.15 (0.11, 0.19)	0.15 (0.11, 0.19)	**0.13 (0.10, 0.18)**
Rhode Island	0.21 (0.18, 0.24)	0.21 (0.18, 0.25)	**0.17 (0.13, 0.21)**
South Caroline	0.14 (0.11, 0.18)	0.14 (0.11, 0.18)	**0.14 (0.10, 0.18)**
South Dakota	0.14 (0.10, 0.18)	0.14 (0.10, 0.18)	**0.12 (0.08, 0.16)**
Tennessee	**0.11 (0.08, 0.15)**	0.11 (0.08, 0.15)	0.11 (0.07, 0.15)
Texas	0.16 (0.13, 0.20)	0.16 (0.13, 0.20)	**0.15 (0.11, 0.19)**
Utah	0.21 (0.17, 0.25)	0.21 (0.18, 0.25)	**0.18 (0.14, 0.23)**
Vermont	0.18 (0.15, 0.22)	0.18 (0.15, 0.22)	**0.15 (0.11, 0.20)**
Virginia	0.13 (0.10, 0.17)	0.13 (0.10, 0.17)	**0.12 (0.08, 0.16)**
Washington	0.18 (0.14, 0.22)	0.18 (0.14, 0.22)	**0.16 (0.12, 0.21)**
West Virginia	0.12 (0.08, 0.15)	0.12 (0.08, 0.15)	**0.11 (0.08, 0.15)**
Wisconsin	0.17 (0.14, 0.22)	0.18 (0.14, 0.22)	**0.16 (0.12, 0.21)**
Wyoming	0.14 (0.11, 0.17)	0.14 (0.11, 0.17)	**0.10 (0.07, 0.14)**

The values in boldface are the selected model. The values in parenthesis after the numbers correspond to the 95% confidence bands of non-rejections values of the integration parameter *d*

**Table 4:** Coefficients in the selected models. Temperature anomaly: results by state

Series	*d* (95% band)	Intercept (*t*-value)	Time trend (*t-*value)	Seasonal AR (*ρ*)
Alabama	0.13 (0.09, 0.17)	–	**–**	0.05945
Arizona	0.17 (0.14, 0.22)	− 0.8528 (− 2.63)	0.0016 (4.69)	− 0.00454
Arkansas	0.11 (0.08, 0.15)	–	–	− 0.00080
California	0.16 (0.13, 0.20)	− 1.0895 (− 3.66)	0.0019 (5.84)	− 0.00195
Colorado	0.13 (0.09, 0.17)	− 1.1094 (− 3.61)	0.0018 (5.47)	0.01285
Connecticut	0.16 (0.12, 0.20)	− 1.4241 (− 3.86)	0.0023 (5.82)	0.00276
Delaware	0.15 (0.11, 0.19)	− 1.2491 (− 3.48)	0.0022 (5.69)	0.01849
Florida	0.18 (0.13, 0.22)	− 0.8277 (− 2.45)	0.00143 (3.89)	0.07951
Georgia	0.14 (0.10, 0.19)	− 0.2558 (− 0.79)	0.00062 (1.76)	0.07171
Idaho	0.16 (0.12, 0.20)	− 0.7570 (− 1.89)	0.00136 (3.11)	0.02073
Illinois	0.11 (0.08, 0.16)	− 0.6431 (− 1.76)	0.00110 (2.74)	− 0.00391
Indiana	0.12 (0.08, 0.16)	− 0.5242 (− 1.39)	0.00100 (2.41)	0.00010
Iowa	0.13 (0.09, 0.17)	− 0.5589 (− 1.26)	0.00092 (1.90)	− 0.00457
Kansas	0.09 (0.06, 0.13)	− 0.6782 (− 2.17)	0.00109 (3.17)	− 0.00023
Kentucky	0.12 (0.08, 0.16)	–	–	0.01729
Louisiana	0.15 (0.11, 0.19)	–	–	0.03604
Maine	0.17 (0.13, 0.22)	− 1.3847 (− 3.43)	0.00227 (5.15)	− 0.04183
Maryland	0.14 (0.10, 0.18)	− 1.0396 (− 3.04)	0.00184 (4.93)	− 0.01524
Massachusetts	0.16 (0.13, 0.20)	− 1.3117 (− 3.57)	0.00224 (5.58)	− 0.00516
Michigan	0.18 (0.14, 0.22)	− 1.3135 (− 2.70)	0.00211 (3.97)	− 0.02393
Minnesota	0.17 (0.12, 0.21)	− 1.2666 (− 2.17)	0.00205 (3.23)	− 0.00762
Mississippi	0.13 (0.10, 0.17)	–	–	0.03484
Missouri	0.10 (0.06, 0.14)	− 0.3617 (− 1.08)	0.00067 (1.81)	0.00618
Montana	0.10 (0.06, 0.14)	− 1.1733 (− 2.99)	0.00182 (4.19)	0.03057
Nebraska	0.09 (0.05, 0.13)	− 0.8014 (− 2.37)	0.00129 (3.44)	0.00683
Nevada	0.15 (0.11, 0.20)	− 0.9589 (− 2.61)	0.00170 (4.23)	− 0.03102
New Hampshire	0.16 (0.13, 0.20)	− 1.2332 (− 3.15)	0.00211 (4.93)	− 0.02735
New Jersey	0.15 (0.12, 0.20)	− 1.5326 (− 4.35)	0.00257 (6.68)	0.01086
New Mexico	0.17 (0.14, 0.21)	− 0.8104 (− 2.64)	0.00153 (4.54)	0.01368
New York	0.15 (0.11, 0.19)	− 0.8596 (− 2.17)	0.00162 (3.75)	− 0.01062
North Caroline	0.13 (0.09, 0.17)	− 0.4588 (− 1.47)	0.00093 (2.71)	0.05030
North Dakota	0.15 (0.11, 0.19)	− 1.5473 (− 2.60)	0.00222 (3.42)	0.03350
Ohio	0.13 (0.09, 0.17)	− 0.6006 (− 1.56)	0.00118 (2.80)	0.00699
Oklahoma	0.10 (0.07, 0.14)	–	–	0.01579
Oregon	0.14 (0.10, 0.19)	− 1.2596 (− 3.90)	0.00192 (5.56)	0.00678
Pennsylvania	0.13 (0.10, 0.18)	− 0.7267 (− 2.09)	0.00137 (3.58)	0.000574
Rhode Island	0.17 (0.13, 0.21)	− 1.5671 (− 4.17)	0.00262 (6.39)	0.00258
South Caroline	0.14 (0.10, 0.18)	− 0.3809 (− 1.16)	0.00081 (2.27)	0.06632
South Dakota	0.12 (0.08, 0.16)	− 1.1157 (− 2.48)	0.00167 (3.27)	0.02146
Tennessee	0.11 (0.08, 0.15)	–	–	0.03163
Texas	0.15 (0.11, 0.19)	− 0.5890 (− 1.86)	0.00104 (3.00)	0.02270
Utah	0.18 (0.14, 0.23)	− 1.3042 (− 3.05)	0.00207 (4.43)	− 0.01792
Vermont	0.15 (0.11, 0.20)	− 1.1418 (− 2.84)	0.00201 (4.56)	− 0.02052
Virginia	0.12 (0.08, 0.16)	− 0.5771 (− 1.87)	0.00111 (3.29)	0.03683
Washington	0.16 (0.12, 0.21)	− 0.8239 (− 2.29)	0.00135 (3.44)	0.03815
West Virginia	0.11 (0.08, 0.15)	− 0.3229 (− 1.00)	0.00076 (2.15)	0.03186
Wisconsin	0.16 (0.12, 0.21)	− 0.8403 (− 1.67)	0.00154 (2.80)	− 0.01586
Wyoming	0.10 (0.07, 0.14)	− 1.2137 (− 3.86)	0.00189 (5.44)	0.01491

**Fig. 4:**
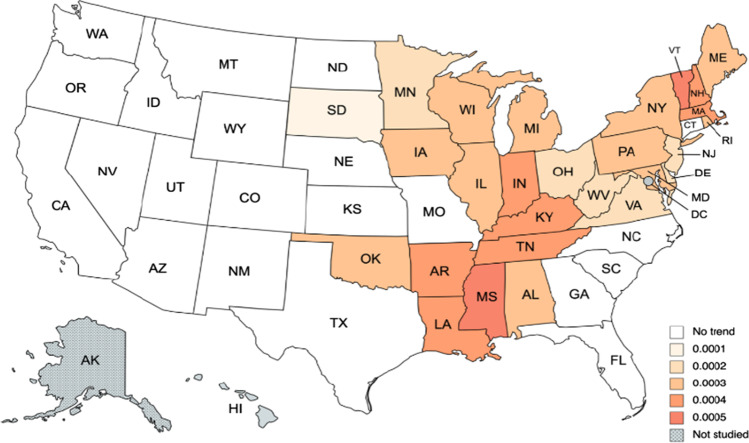
Time trend for precipitation anomaly, based on the results of Table 6

Moving to the precipitation (see, Tables 5 and 6), we first observe that the time trend is now insignificant in a larger number of states, in particular, in 21 states: Arizona, California, Colorado, Connecticut, Florida, Georgia, Idaho, Kansas, Missouri, Montana, Nebraska, Nevada, New Mexico, North Caroline, North Dakota, Oregon, South Caroline, Texas, Utah, Washington, and Wyoming. Among the states with a positive linear time trend, the highest coefficients are observed in Mississippi (0.00047) and Vermont (0.00045) followed by New Hampshire (0.00042), Tennessee, and Louisiana (0.00041). Note that, these states are all in the eastern part of the country (see Fig. 4).

**Table 5:** Estimates of *d* in the precipitation anomaly: results by state

Series	*α* = *β* = 0 in Eq. (1)	*β* = 0 in Eq. (1)	*α* and *β* estimated from the data
Alabama	0.05 (0.01, 0.09)	0.05 (0.01, 0.09)	**0.04 (0.00, 0.09)**
Arizona	**0.11 (0.07, 0.15)**	0.11 (0.07, 0.15)	0.10 (0.07, 0.15)
Arkansas	0.06 (0.02, 0.10)	0.06 (0.02, 0.10)	**0.05 (0.01, 0.10)**
California	**0.09 (0.05, 0.13)**	0.09 (0.05, 0.13)	0.09 (0.05, 0.13)
Colorado	**0.07 (0.03, 0.13)**	0.07 (0.03, 0.12)	0.07 (0.03, 0.12)
Connecticut	**0.03 (− 0.01, 0.07)**	0.03 (− 0.01, 0.07)	0.02 (− 0.01, 0.06)
Delaware	0.04 (0.00, 0.07)	0.04 (0.00, 0.07)	**0.03 (− 0.01, 0.07)**
Florida	**0.06 (0.01, 0.10)**	0.06 (0.01, 0.10)	0.06 (0.01, 0.10)
Georgia	**0.07 (0.03, 0.12)**	0.07 (0.03, 0.12)	0.07 (0.03, 0.12)
Idaho	**0.06 (0.02, 0.11)**	0.06 (0.02, 0.11)	0.06 (0.02, 0.11)
Illinois	0.06 (0.03, 0.11)	0.06 (0.03, 0.11)	**0.05 (0.01, 0.09)**
Indiana	0.06 (0.02, 0.10)	0.06 (0.02, 0.10)	**0.04 (0.00, 0.08)**
Iowa	0.08 (0.04, 0.12)	0.08 (0.04, 0.12)	**0.06 (0.02, 0.11)**
Kansas	**0.08 (0.04, 0.12)**	0.08 (0.04, 0.12)	0.08 (0.03, 0.11)
Kentucky	0.07 (0.03, 0.11)	0.07 (0.03, 0.11)	**0.06 (0.02, 0.10)**
Louisiana	0.06 (0.02, 0.10)	0.06 (0.02, 0.10)	**0.05 (0.01, 0.09)**
Maine	0.04 (0.01, 0.08)	0.04 (0.01, 0.08)	**0.02 (− 0.01, 0.08)**
Maryland	0.04 (0.00, 0.08)	0.04 (0.00, 0.08)	**0.03 (− 0.01, 0.07)**
Massachusetts	0.03 (0.00, 0.08)	0.04 (0.00, 0.07)	**0.01 (− 0.02, 0.05)**
Michigan	0.04 (0.01, 0.07)	0.04 (0.01, 0.07)	**0.00 (− 0.04, 0.04)**
Minnesota	0.07 (0.03, 0.10)	0.07 (0.03, 0.10)	**0.05 (0.01, 0.09)**
Mississippi	0.07 (0.03, 0.11)	0.07 (0.03, 0.11)	**0.05 (0.02, 0.10)**
Missouri	**0.07 (0.03, 0.11)**	0.07 (0.03, 0.11)	0.07 (0.03, 0.11)
Montana	**0.04 (0.00, 0.08)**	0.04 (0.00, 0.08)	0.04 (0.00, 0.08)
Nebraska	**0.06 (0.03, 0.10)**	0.06 (0.03, 0.10)	0.06 (0.02, 0.10)
Nevada	**0.08 (0.04, 0.12)**	0.08 (0.04, 0.12)	0.08 (0.04, 0.12)
New Hampshire	0.03 (0.00, 0.06)	0.03 (0.00, 0.06)	**0.00 (− 0.04, 0.03)**
New Jersey	0.04 (0.01, 0.08)	0.04 (0.01, 0.08)	**0.04 (0.00, 0.08)**
New Mexico	**0.09 (0.05, 0.13)**	0.09 (0.05, 0.13)	0.09 (0.05, 0.13)
New York	0.05 (0.02, 0.09)	0.05 (0.02, 0.09)	**0.03 (− 0.01, 0.07)**
North Caroline	**0.05 (0.01, 0.09)**	0.05 (0.01, 0.09)	0.05 (0.01, 0.09)
North Dakota	**0.04 (0.00, 0.08)**	0.04 (0.00, 0.08)	0.03 (− 0.01, 0.07)
Ohio	0.05 (0.02, 0.09)	0.05 (0.02, 0.09)	**0.05 (0.01, 0.09)**
Oklahoma	0.07 (0.04, 0.11)	0.07 (0.04, 0.11)	**0.06 (0.02, 0.10)**
Oregon	**0.06 (0.02, 0.10)**	0.06 (0.02, 0.10)	0.06 (0.02, 0.10)
Pennsylvania	0.05 (0.02, 0.09)	0.05 (0.02, 0.09)	**0.04 (0.01, 0.08)**
Rhode Island	0.03 (− 0.01, 0.06)	0.03 (− 0.01, 0.06)	**0.01 (− 0.02, 0.05)**
South Caroline	**0.05 (0.01, 0.09)**	0.05 (0.01, 0.09)	0.05 (0.01, 0.09)
South Dakota	0.08 (0.04, 0.11)	0.08 (0.04, 0.11)	**0.07 (0.03, 0.11)**
Tennessee	0.06 (0.02, 0.10)	0.06 (0.02, 0.10)	**0.05 (0.01, 0.09)**
Texas	**0.12 (0.09, 0.17)**	0.12 (0.09, 0.17)	0.12 (0.08, 0.17)
Utah	**0.08 (0.05, 0.12)**	0.08 (0.05, 0.12)	0.08 (0.05, 0.12)
Vermont	0.05 (0.02, 0.08)	0.05 (0.02, 0.08)	**0.01 (− 0.03, 0.05)**
Virginia	0.06 (0.02, 0.10)	0.06 (0.02, 0.10)	**0.05 (0.01, 0.09)**
Washington	**0.05 (0.01, 0.09)**	0.05 (0.01, 0.09)	0.05 (0.01, 0.09)
West Virginia	0.06 (0.02, 0.10)	0.06 (0.02, 0.10)	**0.05 (0.02, 0.09)**
Wisconsin	0.05 (0.02, 0.09)	0.05 (0.02, 0.09)	**0.04 (0.00, 0.08)**
Wyoming	**0.06 (0.02, 0.10)**	0.06 (0.02, 0.10)	0.06 (0.02, 0.10)

The values in boldface are the selected model. The values in parenthesis after the numbers correspond to the 95% confidence bands of non-rejections values of the integration parameter *d*

**Table 6:** Selected coefficients in the precipitation anomaly: results by state

Series	*d* (95% band)	An intercept	An intercept and time trend	Seasonal AR (*ρ*)
Alabama	0.04 (0.00, 0.09)	− 0.1503 (− 1.13)	0.00026 (1.66)	0.01124
Arizona	0.11 (0.07, 0.15)	–	–	− 0.03653
Arkansas	0.05 (0.01, 0.10)	− 0.2280 (− 1.66)	0.00035 (2.33)	0.01403
California	0.09 (0.05, 0.13)	–	–	0.03145
Colorado	0.07 (0.03, 0.13)	–	–	− 0.00510
Connecticut	0.03 (− 0.01, 0.07)	–	–	− 0.02966
Delaware	0.03 (− 0.01, 0.07)	− 0.1290 (− 1.26)	0.00021 (1.86)	− 0.05769
Florida	0.06 (0.01, 0.10)	–	–	− 0.01384
Georgia	0.07 (0.03, 0.12)	–	–	− 0.01317
Idaho	0.06 (0.02, 0.11)	–	–	− 0.01912
Illinois	0.05 (0.01, 0.09)	− 0.2070 (− 2.14)	0.00034 (3.15)	− 0.00571
Indiana	0.04 (0.00, 0.08)	− 0.2138 (− 2.29)	0.00037 (2.76)	− 0.00137
Iowa	0.06 (0.02, 0.11)	− 0.1776 (− 1.86)	0.00029 (2.76)	0.00860
Kansas	0.08 (0.04, 0.12)	–	–	− 0.01562
Kentucky	0.06 (0.02, 0.10)	− 0.2024 (− 1.66)	0.00036 (2.65)	0.00929
Louisiana	0.05 (0.01, 0.09)	− 0.2800 (− 1.84)	0.00041 (2.45)	0.00837
Maine	0.02 (− 0.01, 0.08)	− 0.1801 (− 2.26)	0.00031 (3.50)	− 0.02164
Maryland	0.03 (− 0.01, 0.07)	− 0.1594 (− 1.68)	0.00026 (2.41)	− 0.04158
Massachusetts	0.01 (− 0.02, 0.05)	− 0.2328 (− 2.51)	0.00040 (3.86)	− 0.04972
Michigan	0.00 (− 0.04, 0.04)	− 0.1827 (− 3.90)	0.00029 (5.62)	0.04077
Minnesota	0.05 (0.01, 0.09)	− 0.1083 (− 1.66)	0.00019 (2.61)	0.03948
Mississippi	0.05 (0.02, 0.10)	− 0.3130 (− 2.18)	0.00047 (2.94)	0.01155
Missouri	0.07 (0.03, 0.11)	–	–	− 0.01792
Montana	0.04 (0.00, 0.08)	–	–	0.01451
Nebraska	0.06 (0.03, 0.10)	–	–	0.00346
Nevada	0.08 (0.04, 0.12)	–	–	− 0.03461
New Hampshire	0.00 (− 0.04, 0.03)	− 0.2419 (− 3.05)	0.00042 (4.63)	− 0.01772
New Jersey	0.04 (0.00, 0.08)	− 0.1167 (− 1.06)	0.00023 (1.85)	− 0.03395
New Mexico	0.09 (0.05, 0.13)	–	–	− 0.00669
New York	0.03 (− 0.01, 0.07)	− 0.1849 (− 2.58)	0.00032 (3.94)	− 0.00075
North Caroline	0.05 (0.01, 0.09)	–	–	− 0.03856
North Dakota	0.04 (0.00, 0.08)	–	–	− 0.07774
Ohio	0.05 (0.01, 0.09)	− 0.1187 (− 1.36)	0.00023 (2.34)	− 0.00685
Oklahoma	0.06 (0.02, 0.10)	− 0.2026 (− 1.84)	0.00028 (2.30)	− 0.02971
Oregon	0.06 (0.02, 0.10)	–	–	− 0.04276
Pennsylvania	0.04 (0.01, 0.08)	− 0.1502 (− 1.77)	0.00027 (2.81)	− 0.00566
Rhode Island	0.01 (− 0.02, 0.05)	− 0.1676 (− 1.65)	0.00033 (2.85)	− 0.04291
South Caroline	0.05 (0.01, 0.09)	–	–	− 0.01514
South Dakota	0.07 (0.03, 0.11)	− 0.0718 (− 1.13)	0.00013 (1.78)	0.00306
Tennessee	0.05 (0.01, 0.09)	− 0.2537 (− 2.02)	0.00041 (2.90)	0.02049
Texas	0.12 (0.09, 0.17)	–	–	0.01306
Utah	0.08 (0.05, 0.12)	–	–	− 0.02521
Vermont	0.01 (− 0.03, 0.05)	− 0.2800 (− 3.74)	0.00045 (5.37)	− 0.01559
Virginia	0.05 (0.01, 0.09)	− 0.1288 (− 1.28)	0.00023 (2.10)	− 0.04226
Washington	0.05 (0.01, 0.09)	–	–	− 0.00965
West Virginia	0.05 (0.02, 0.09)	− 0.0889 (− 0.96)	0.00017 (1.65)	− 0.01751
Wisconsin	0.04 (0.00, 0.08)	− 0.1503 (− 2.09)	0.00025 (3.07)	0.04416
Wyoming	0.06 (0.02, 0.10)	–	–	− 0.00671

**Fig. 5:**
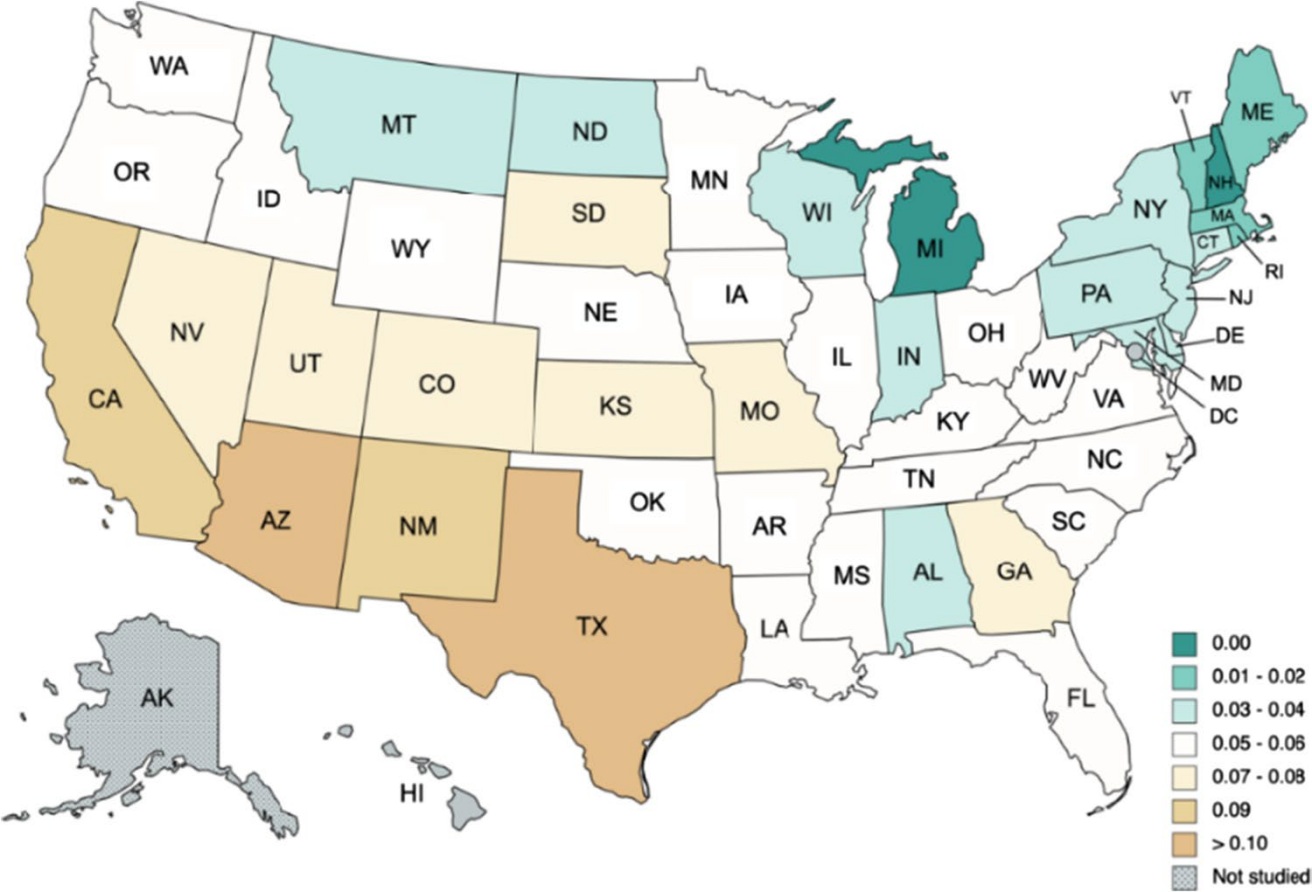
Estimate of *d* for precipitation anomaly, based on the results of Table 6

With respect to the degree of integration, we see that the estimated values of the integration order d are smaller than those of the temperatures: short memory or I(0) behaviour cannot be rejected in 14 states: Alabama, Indiana, North Dakota and Wisconsin (with *d* = 0.04); Connecticut, Delaware, Maryland and New York (0.03), Maine (0.02), Massachusetts, Rhode Island and Vermont (*d* = 0.01), and Michigan and New Hampshire (*d* = 0.00); for the rest of the states, the estimate of d is significantly higher than 0, implying a long memory pattern, and the highest values are obtained at Arizona (*d* = 0.11) and Texas (0.12). Figure 5 provides a graphical summary of the results relating the differencing parameter. We observe that the states with the highest degrees of persistence seems to be located in the Southwest, while those with the lowest values are in the Northeast. As with temperature anomaly, these findings of higher persistence in precipitation anomaly seem to be negatively correlated with climate change–related risks and degree of preparedness, albeit in a tentative manner.

## Discussion and conclusions

The time series features of the temperature and precipitation anomalies in the US have been examined in this paper, looking first at the aggregated, data and then at the data disaggregated by the 40 contiguous states. In order to do that, we have employed techniques based on fractional differentiation allowing thus the number of differences that can be used in the series to take a fractional value.

Starting with the aggregated data, our results support the hypothesis of long memory or strong dependence since the differentiation order is significantly positive in the two cases of temperature and precipitation anomalies, and the time trend coefficient is positive in the two cases, with it being higher for the temperatures.

If we look at the data disaggregated by states, starting with the temperature anomaly, we see that the coefficient for the time trend is significantly positive in the majority of the states, barring seven cases with the insignificant trend, and they being all located in the South East. For the estimate of the differencing parameter, there is a large degree of heterogeneity across the states, with the value of d ranging from 0.09 (Nebraska and Kansas) and 0.10 (Missouri, Montana, Oklahoma, and Wyoming) to 0.18 in Florida and Michigan.

For the precipitation anomaly, the trend is now found to be statistically insignificant in a large number of states, and the degree of differentiation is slightly smaller than for the temperature anomaly. In fact, the hypothesis of a short memory pattern (i.e., *d* = 0) cannot be rejected in fourteen states and the highest degree of integration is observed in Arizona (*d* = 0.11) and Texas (0.12).

Climate risks, as captured by the behavior of temperature and precipitation anomalies, are known to have an effect on economic activity (Descêhnes and Greenstone 2007; Dell et al. 2009, 2012, 2014). Our results suggest that given the heterogeneity in terms of the trend and persistence of temperature and precipitation anomalies, the nature and strength of policies adopted by the local governments to mitigate climate change would need to be different from one another; i.e., state-specific policies need to be pursued to accurately tackle the issue of local climate change. At the same time, we must also emphasize that policymakers must not rely on aggregate results to come up with policy decisions at the state level.

Given that, climate risks have also been associated with the volatility of temperature and precipitation anomalies (Donadelli et al. 2017, Donadelli et al. 2021a, 2021b, 2021c; Kotz et al. 2021), as part of future research, it would be interesting to conduct similar analyses on the assessment of trend and persistence of the variance of these two series. From a methodological viewpoint, future research might investigate the presence of non-linear and/or cyclical structures in the data still in the context of fractional integration.

## Data Availability

Data are available from the authors upon request.

## Footnotes

1 See: https://www.ncdc.noaa.gov/cag/national/time-series and https://www.ncdc.noaa.gov/cag/statewide/time-series.

2 Since we do not have a time series available for this index, to which ideally, we would have wanted to relate a time series of the persistence derived from a time-varying estimation of our underlying ARFIMA model, we cannot perform a proper regression-based analysis. Hence, we are speculating based on a one-shot evidence, i.e., the heat map provided by Policygenius, and the reader must be cautious about our conclusions drawn in this regard, as a result of which, we only call such a correlation as “tentative”. Understandably, more detailed analysis is required in the future along this question of persistence and climate change risks and preparedness of US states.

3 The factors that went into this analysis were grouped into the following five categories: (1) Drought: The likelihood of negative economic impacts as a result of drought, the number of people and fresh water sources exposed, and the state’s ability to recover or adapt to a future with drought; (2) Extreme heat: Projected number of dangerously hot days by 2050 and the percentage of people vulnerable to dangerously high temperatures; (3) Wildfires: Fraction of housing units directly or indirectly exposed to wildfire, as well as wildfire likelihood and home susceptibility; (4) Flooding: Percentage of people living in 100 and 500-year floodplains, projected 30-year increase in number of properties with flood risk, and the percentage of the population living in a 100-year coastal floodplain, and; (5) Climate change preparedness: How well each state is responding to current and future climate change threats.

## Code availability (software application or custom code)

Codes are available from the authors upon request.
